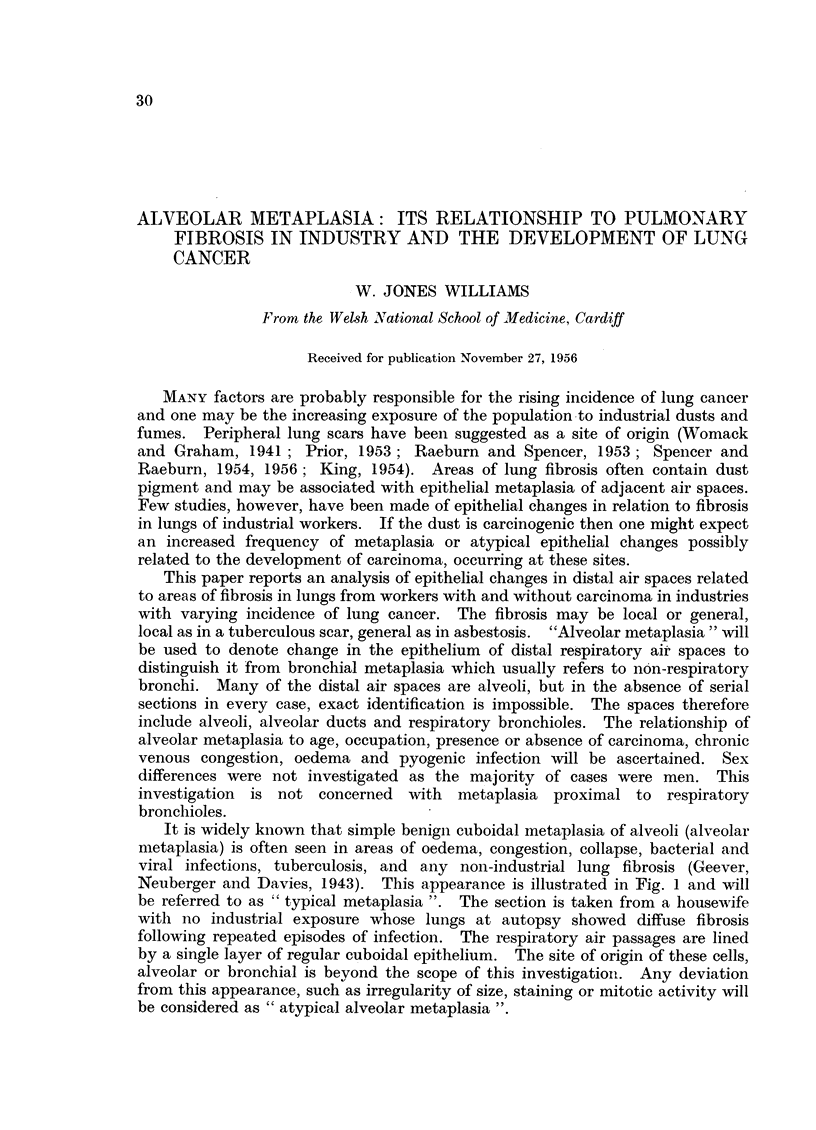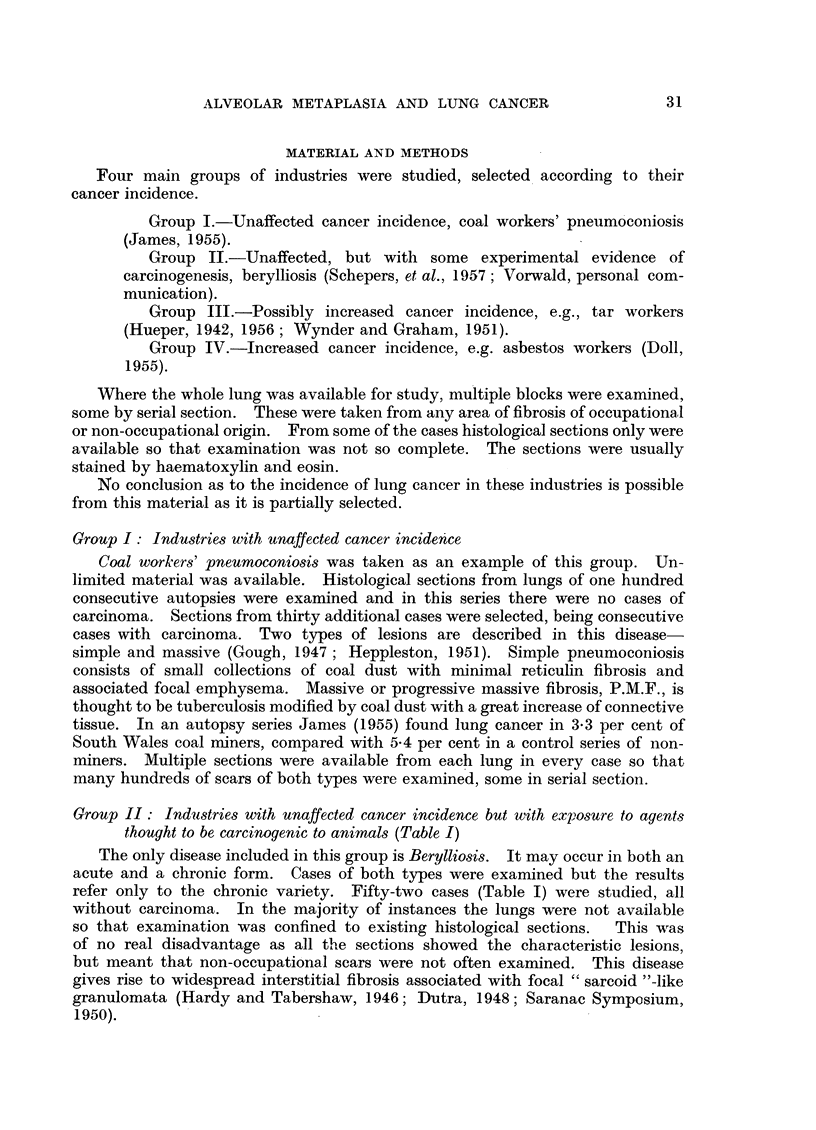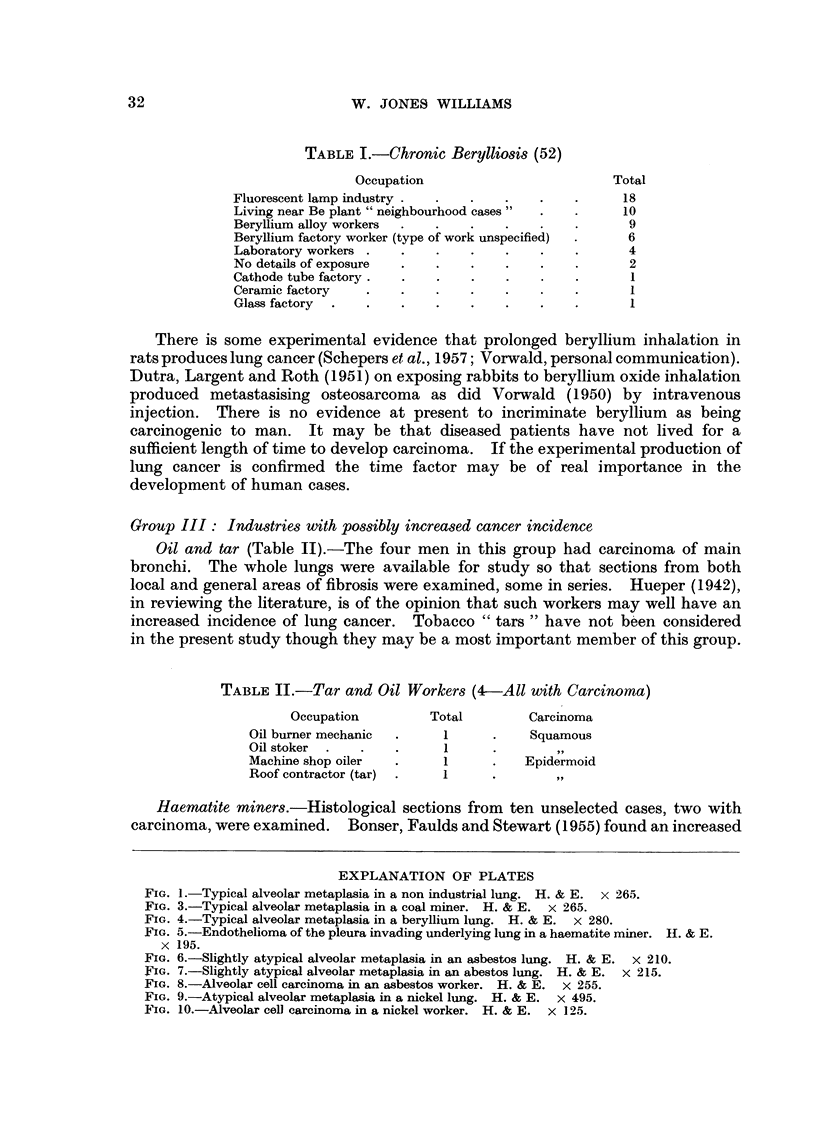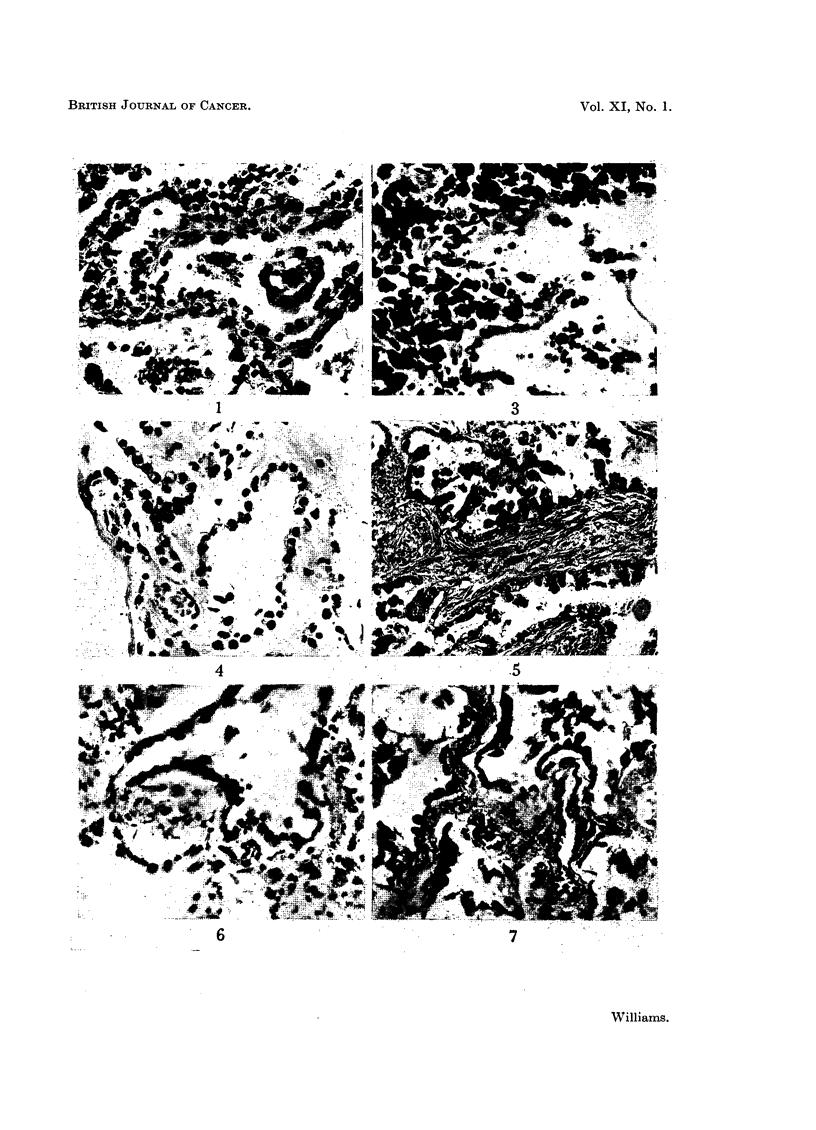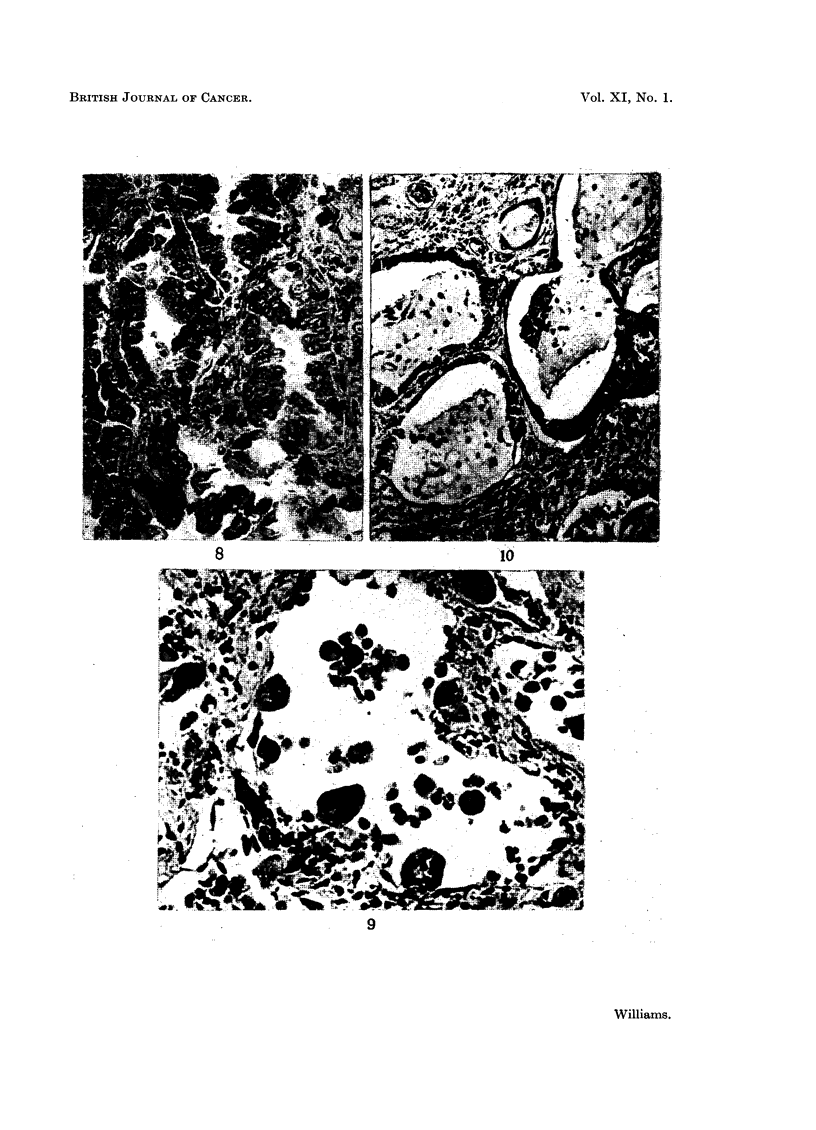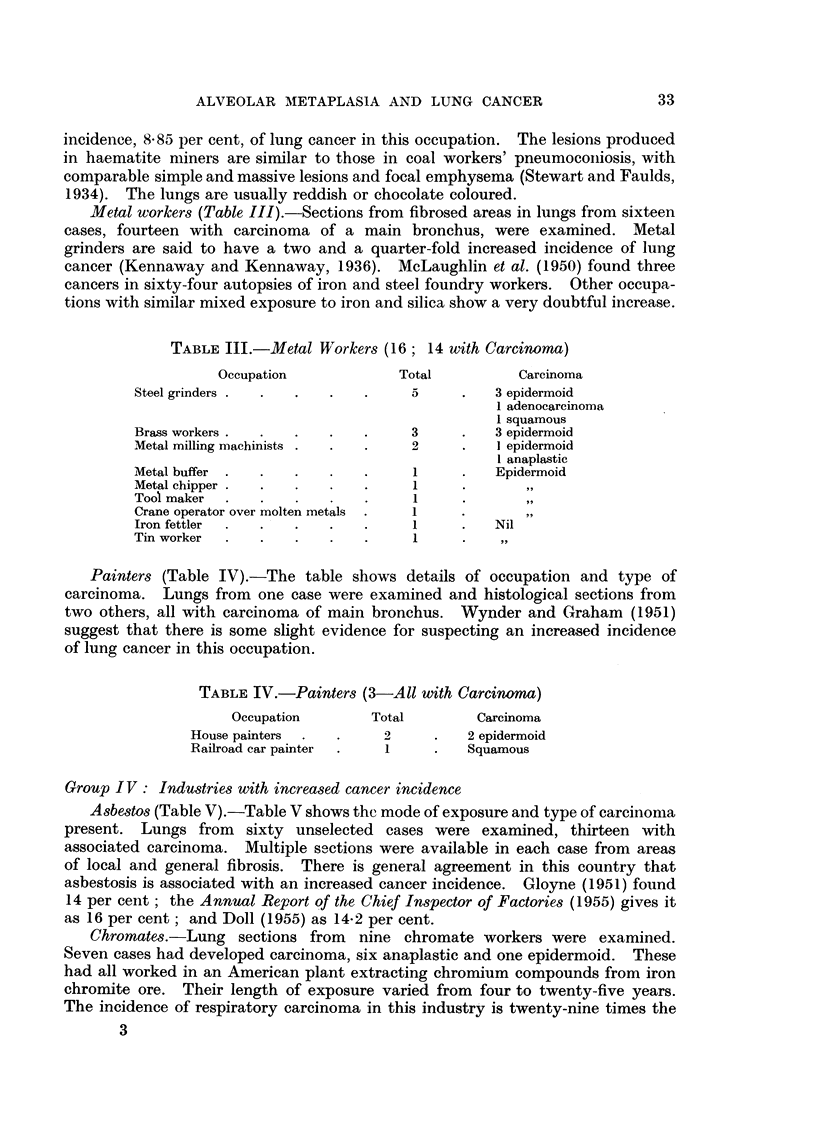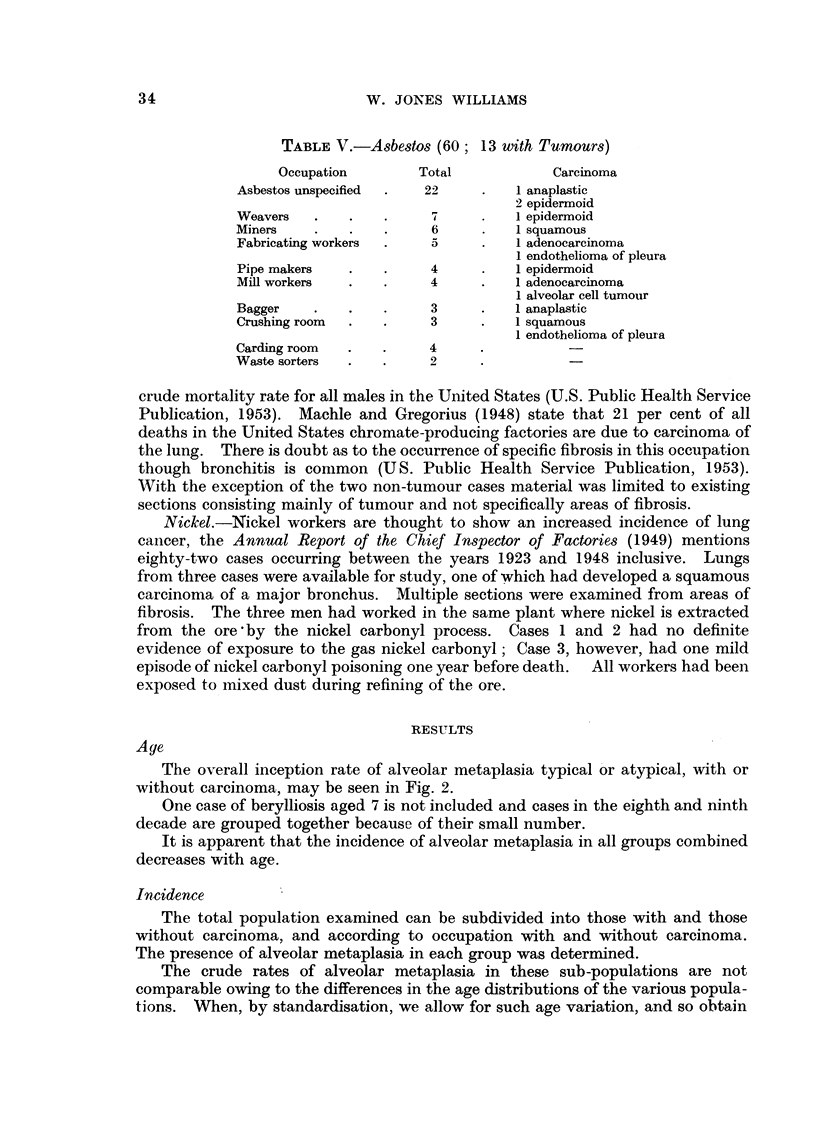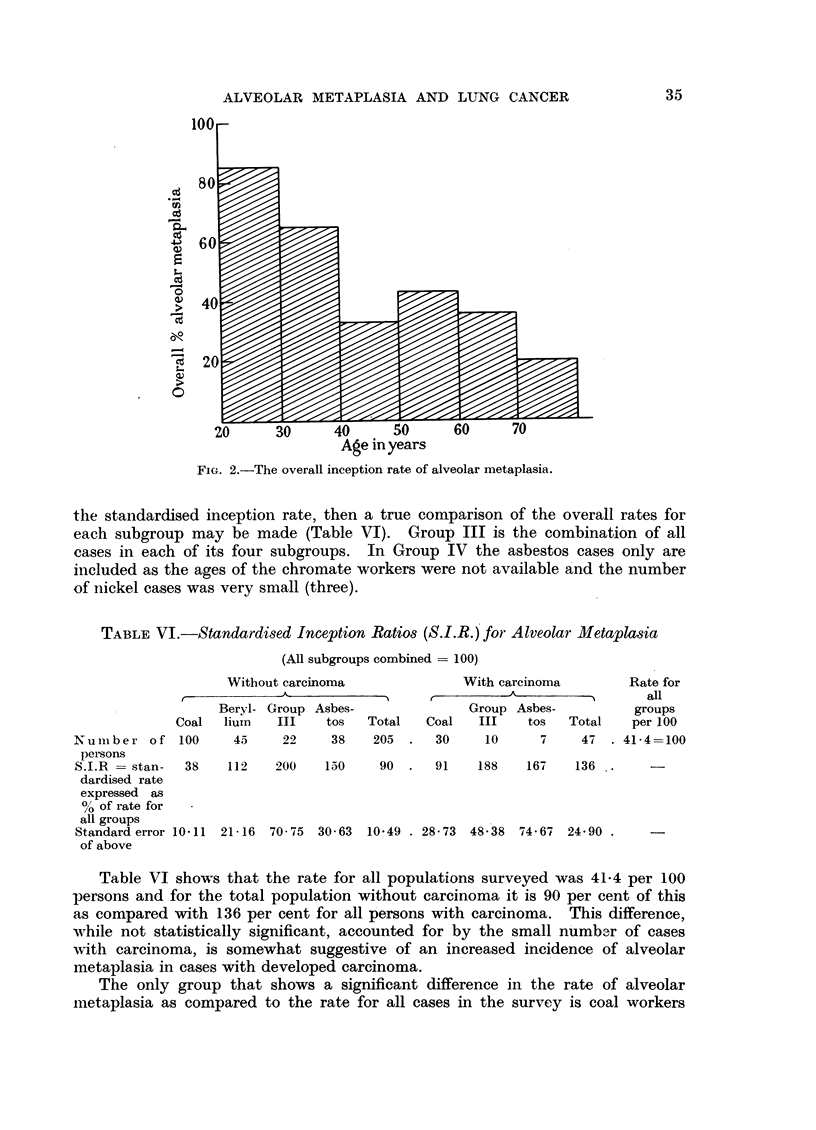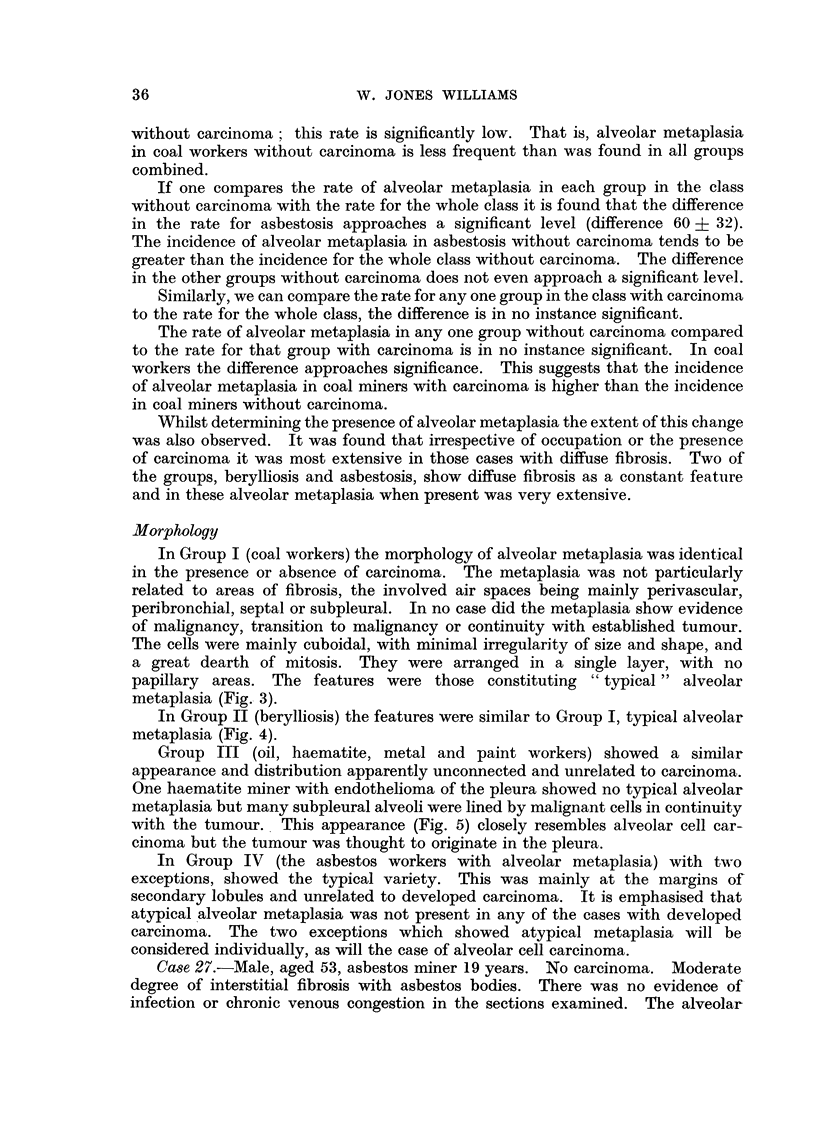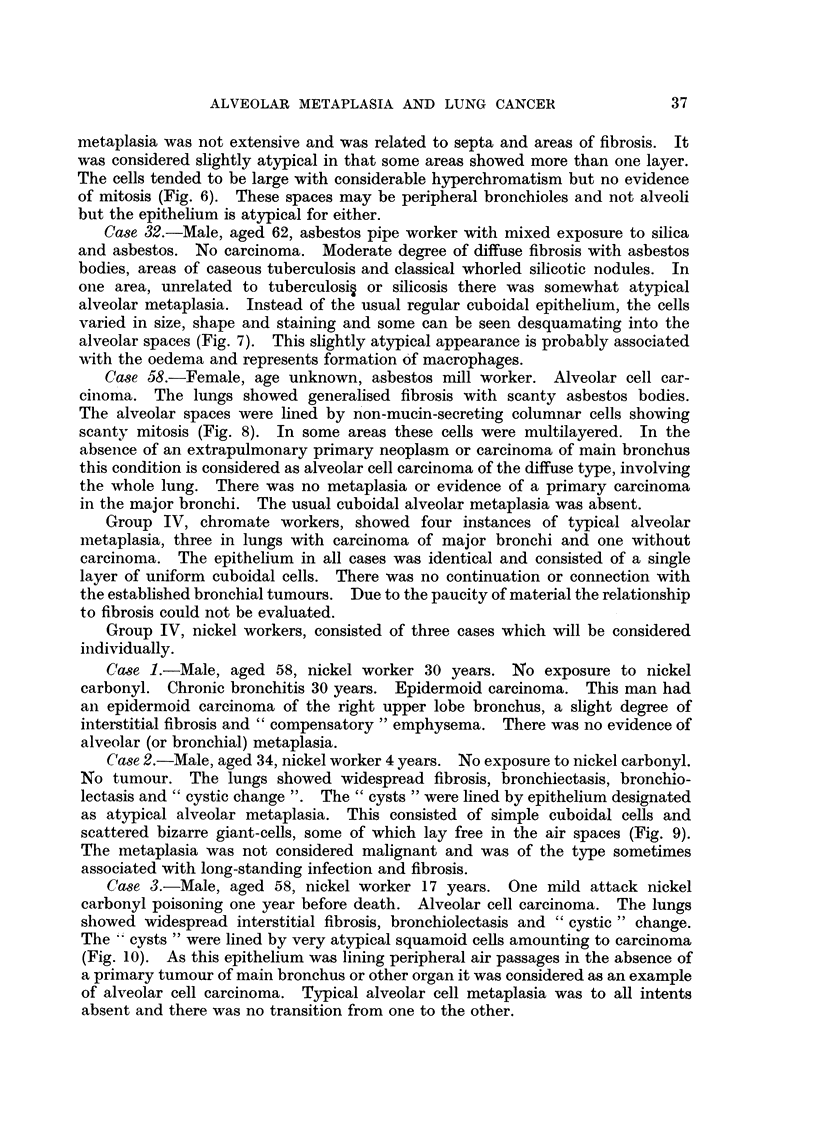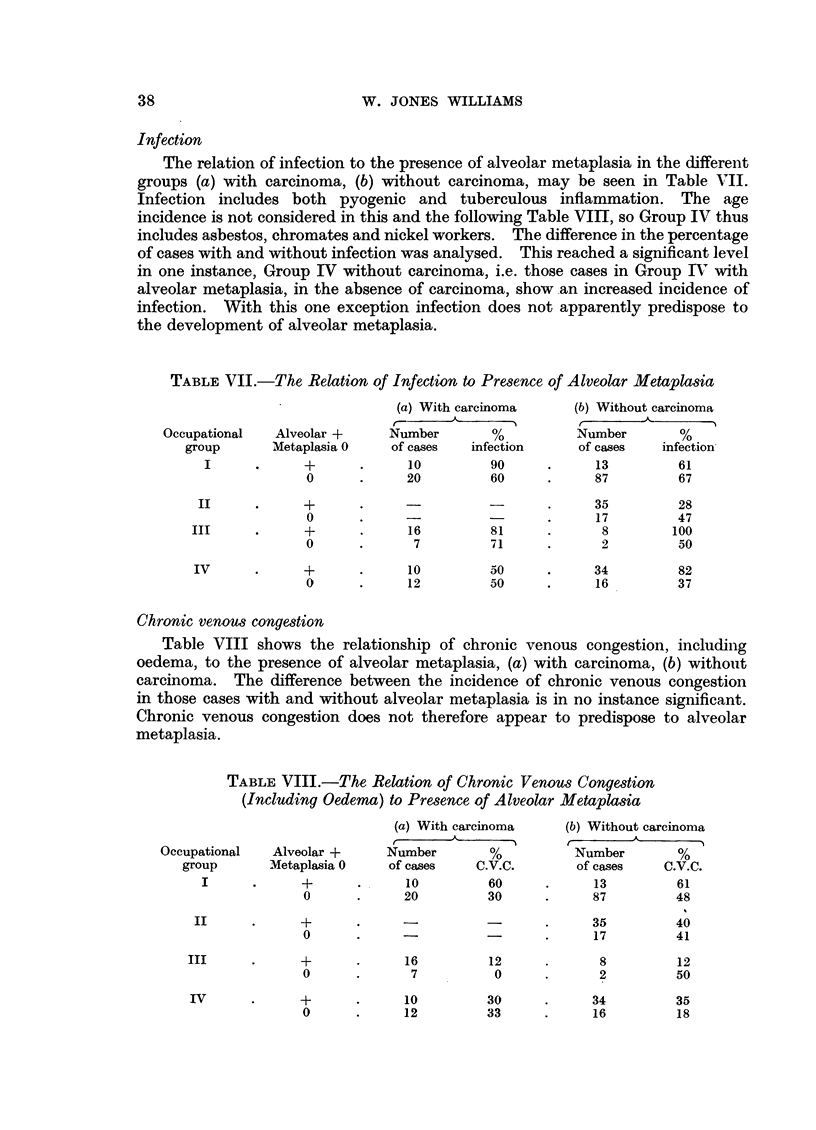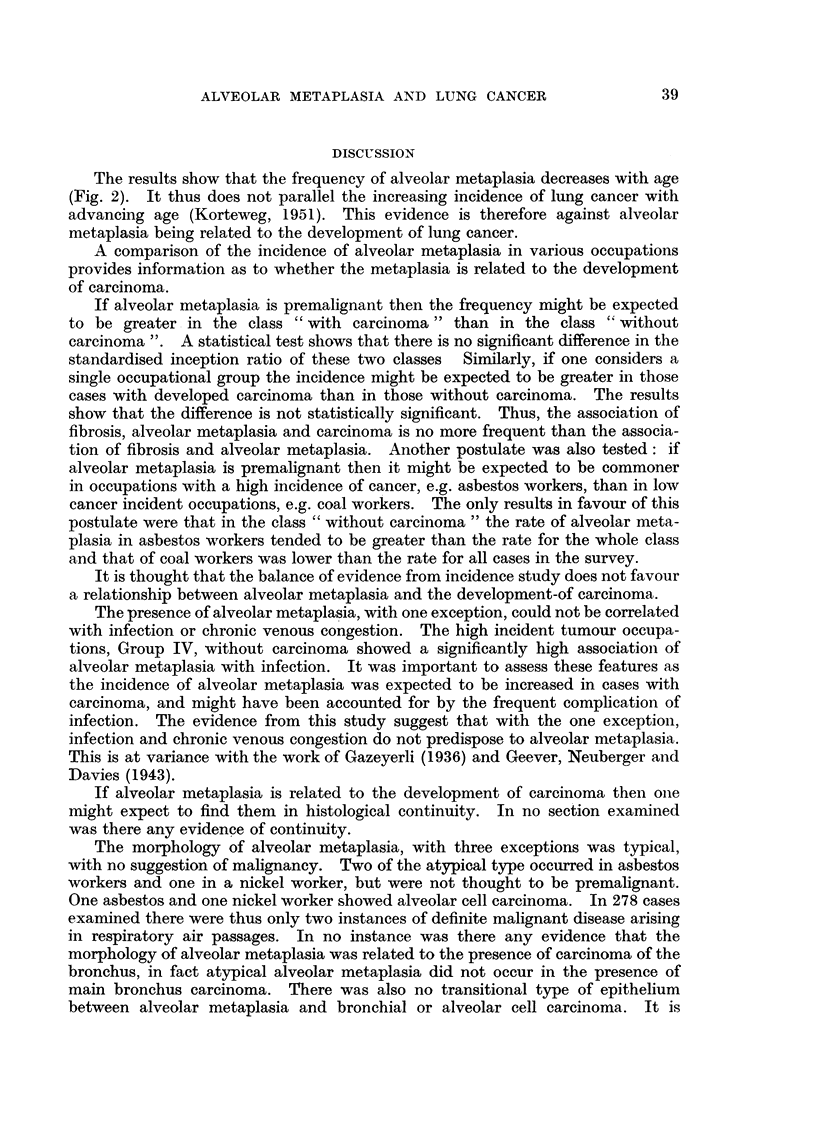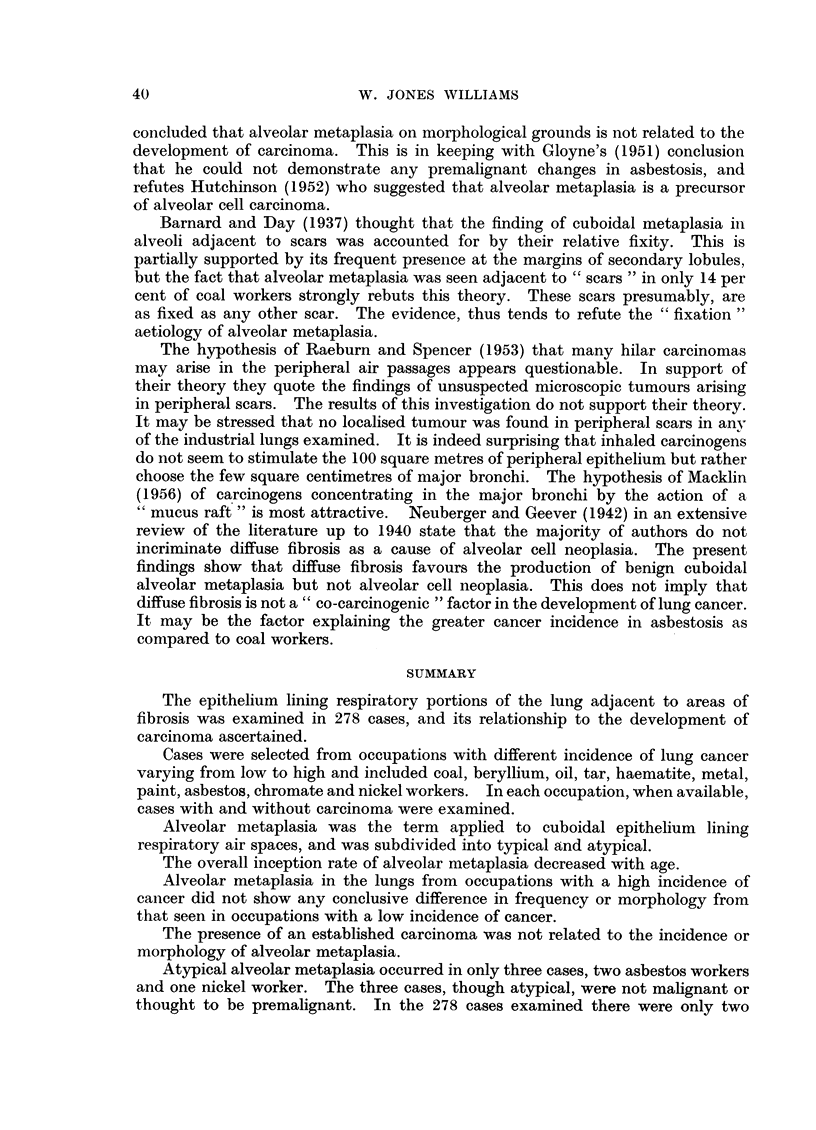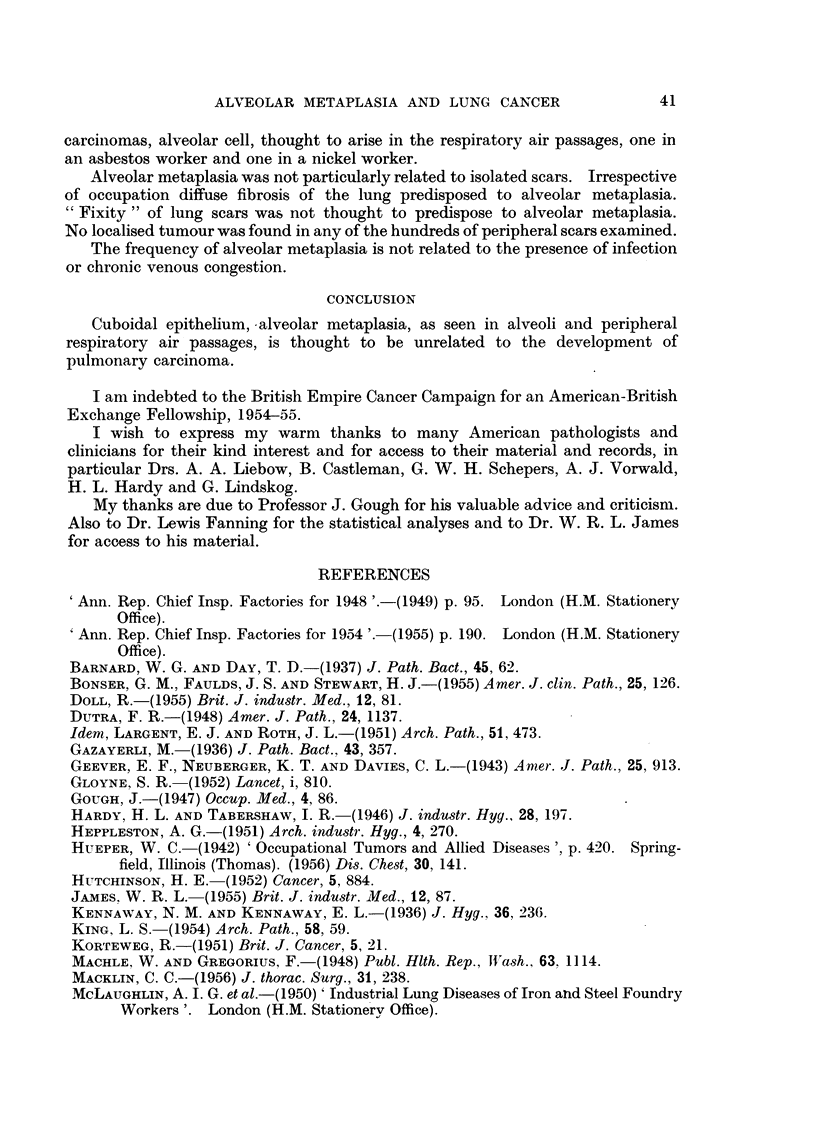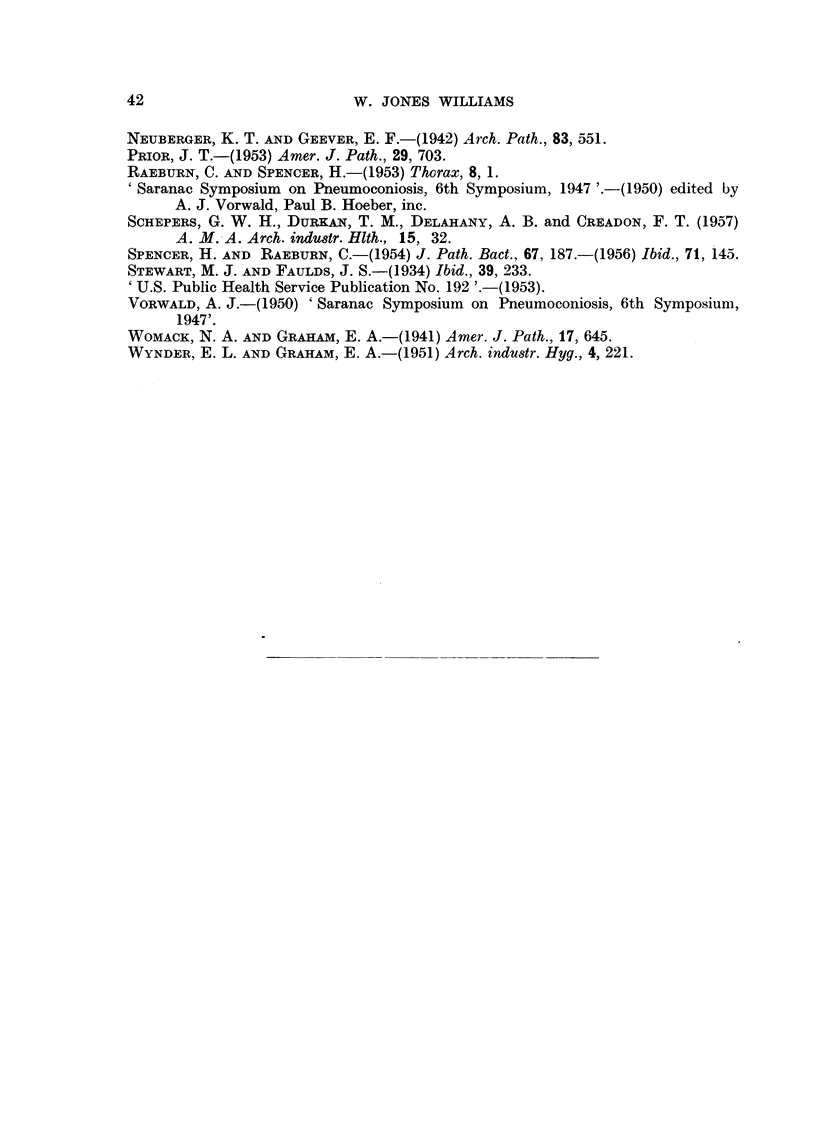# Alveolar Metaplasia: Its Relationship to Pulmonary Fibrosis in Industry and the Development of Lung Cancer

**DOI:** 10.1038/bjc.1957.5

**Published:** 1957-03

**Authors:** W. Jones Williams

## Abstract

**Images:**


### ALVEOLAR METAPLASIA: ITS RELATIONSHIP TO PULMONARY FIBROSIS IN INDUSTRY AND THE DEVELOPMENT OF LUNG CANCER

W. JONES WILLIAMS

*From the Welsh National School of Medicine, Cardiff*

Received for publication November 27, 1956

MANY factors are probably responsible for the rising incidence of lung cancer and one may be the increasing exposure of the population to industrial dusts and fumes. Peripheral lung scars have been suggested as a site of origin (Womack and Graham, 1941; Prior, 1953; Raeburn and Spencer, 1953; Spencer and Raeburn, 1954, 1956; King, 1954). Areas of lung fibrosis often contain dust pigment and may be associated with epithelial metaplasia of adjacent air spaces. Few studies, however, have been made of epithelial changes in relation to fibrosis in lungs of industrial workers. If the dust is carcinogenic then one might expect an increased frequency of metaplasia or atypical epithelial changes possibly related to the development of carcinoma, occurring at these sites.

This paper reports an analysis of epithelial changes in distal air spaces related to areas of fibrosis in lungs from workers with and without carcinoma in industries with varying incidence of lung cancer. The fibrosis may be local or general, local as in a tuberculous scar, general as in asbestosis. "Alveolar metaplasia" will be used to denote change in the epithelium of distal respiratory air spaces to distinguish it from bronchial metaplasia which usually refers to non-respiratory bronchi. Many of the distal air spaces are alveoli, but in the absence of serial sections in every case, exact identification is impossible. The spaces therefore include alveoli, alveolar ducts and respiratory bronchioles. The relationship of alveolar metaplasia to age, occupation, presence or absence of carcinoma, chronic venous congestion, oedema and pyogenic infection will be ascertained. Sex differences were not investigated as the majority of cases were men. This investigation is not concerned with metaplasia proximal to respiratory bronchioles.

It is widely known that simple benign cuboidal metaplasia of alveoli (alveolar metaplasia) is often seen in areas of oedema, congestion, collapse, bacterial and viral infections, tuberculosis, and any non-industrial lung fibrosis (Geever, Neuberger and Davies, 1943). This appearance is illustrated in Fig. 1 and will be referred to as "typical metaplasia". The section is taken from a housewife with no industrial exposure whose lungs at autopsy showed diffuse fibrosis following repeated episodes of infection. The respiratory air passages are lined by a single layer of regular cuboidal epithelium. The site of origin of these cells, alveolar or bronchial is beyond the scope of this investigation. Any deviation from this appearance, such as irregularity of size, staining or mitotic activity will be considered as "atypical alveolar metaplasia".

### MATERIAL AND METHODS

Four main groups of industries were studied, selected according to their cancer incidence.

Group I.—Unaffected cancer incidence, coal workers' pneumoconiosis (James, 1955).

Group II.—Unaffected, but with some experimental evidence of carcinogenesis, berylliosis (Schepers, *et al.*, 1957 ; Vorwald, personal communication).

Group III.—Possibly increased cancer incidence, e.g., tar workers (Hueper, 1942, 1956 ; Wynder and Graham, 1951).

Group IV.—Increased cancer incidence, e.g. asbestos workers (Doll, 1955).

Where the whole lung was available for study, multiple blocks were examined, some by serial section. These were taken from any area of fibrosis of occupational or non-occupational origin. From some of the cases histological sections only were available so that examination was not so complete. The sections were usually stained by haematoxylin and eosin.

No conclusion as to the incidence of lung cancer in these industries is possible from this material as it is partially selected.

*Group I : Industries with unaffected cancer incidence*

*Coal workers' pneumoconiosis* was taken as an example of this group. Unlimited material was available. Histological sections from lungs of one hundred consecutive autopsies were examined and in this series there were no cases of carcinoma. Sections from thirty additional cases were selected, being consecutive cases with carcinoma. Two types of lesions are described in this disease—simple and massive (Gough, 1947 ; Heppleston, 1951). Simple pneumoconiosis consists of small collections of coal dust with minimal reticulin fibrosis and associated focal emphysema. Massive or progressive massive fibrosis, P.M.F., is thought to be tuberculosis modified by coal dust with a great increase of connective tissue. In an autopsy series James (1955) found lung cancer in 3·3 per cent of South Wales coal miners, compared with 5·4 per cent in a control series of non-miners. Multiple sections were available from each lung in every case so that many hundreds of scars of both types were examined, some in serial section.

*Group II : Industries with unaffected cancer incidence but with exposure to agents thought to be carcinogenic to animals (Table I)*

The only disease included in this group is *Berylliosis.* It may occur in both an acute and a chronic form. Cases of both types were examined but the results refer only to the chronic variety. Fifty-two cases (Table I) were studied, all without carcinoma. In the majority of instances the lungs were not available so that examination was confined to existing histological sections. This was of no real disadvantage as all the sections showed the characteristic lesions, but meant that non-occupational scars were not often examined. This disease gives rise to widespread interstitial fibrosis associated with focal " sarcoid "-like granulomata (Hardy and Tabershaw, 1946 ; Dutra, 1948 ; Saranac Symposium, 1950).

TABLE I.—*Chronic Berylliosis* (52)

| Occupation | Total |
|---|---|
| Fluorescent lamp industry | 18 |
| Living near Be plant " neighbourhood cases " | 10 |
| Beryllium alloy workers | 9 |
| Beryllium factory worker (type of work unspecified) | 6 |
| Laboratory workers | 4 |
| No details of exposure | 2 |
| Cathode tube factory | 1 |
| Ceramic factory | 1 |
| Glass factory | 1 |

There is some experimental evidence that prolonged beryllium inhalation in rats produces lung cancer (Schepers *et al.*, 1957; Vorwald, personal communication). Dutra, Largent and Roth (1951) on exposing rabbits to beryllium oxide inhalation produced metastasising osteosarcoma as did Vorwald (1950) by intravenous injection. There is no evidence at present to incriminate beryllium as being carcinogenic to man. It may be that diseased patients have not lived for a sufficient length of time to develop carcinoma. If the experimental production of lung cancer is confirmed the time factor may be of real importance in the development of human cases.

*Group III : Industries with possibly increased cancer incidence*

*Oil and tar* (Table II).—The four men in this group had carcinoma of main bronchi. The whole lungs were available for study so that sections from both local and general areas of fibrosis were examined, some in series. Hueper (1942), in reviewing the literature, is of the opinion that such workers may well have an increased incidence of lung cancer. Tobacco " tars " have not been considered in the present study though they may be a most important member of this group.

TABLE II.—*Tar and Oil Workers* (4—*All with Carcinoma*)

| Occupation | Total | Carcinoma |
|---|---|---|
| Oil burner mechanic | 1 | Squamous |
| Oil stoker | 1 | ,, |
| Machine shop oiler | 1 | Epidermoid |
| Roof contractor (tar) | 1 | ,, |

*Haematite miners.*—Histological sections from ten unselected cases, two with carcinoma, were examined. Bonser, Faulds and Stewart (1955) found an increased

EXPLANATION OF PLATES

FIG. 1.—Typical alveolar metaplasia in a non industrial lung. H. & E. × 265.
FIG. 3.—Typical alveolar metaplasia in a coal miner. H. & E. × 265.
FIG. 4.—Typical alveolar metaplasia in a beryllium lung. H. & E. × 280.
FIG. 5.—Endothelioma of the pleura invading underlying lung in a haematite miner. H. & E. × 195.
FIG. 6.—Slightly atypical alveolar metaplasia in an asbestos lung. H. & E. × 210.
FIG. 7.—Slightly atypical alveolar metaplasia in an abestos lung. H. & E. × 215.
FIG. 8.—Alveolar cell carcinoma in an asbestos worker. H. & E. × 255.
FIG. 9.—Atypical alveolar metaplasia in a nickel lung. H. & E. × 495.
FIG. 10.—Alveolar cell carcinoma in a nickel worker. H. & E. × 125.

1

3

4

5

6

7

8 10 9

Williams.

incidence, 8·85 per cent, of lung cancer in this occupation. The lesions produced in haematite miners are similar to those in coal workers' pneumoconiosis, with comparable simple and massive lesions and focal emphysema (Stewart and Faulds, 1934). The lungs are usually reddish or chocolate coloured.

*Metal workers* (*Table III*).—Sections from fibrosed areas in lungs from sixteen cases, fourteen with carcinoma of a main bronchus, were examined. Metal grinders are said to have a two and a quarter-fold increased incidence of lung cancer (Kennaway and Kennaway, 1936). McLaughlin *et al.* (1950) found three cancers in sixty-four autopsies of iron and steel foundry workers. Other occupations with similar mixed exposure to iron and silica show a very doubtful increase.

TABLE III.—*Metal Workers* (16 ; 14 *with Carcinoma*)

| Occupation | Total | Carcinoma |
|---|---|---|
| Steel grinders | 5 | 3 epidermoid<br>1 adenocarcinoma<br>1 squamous |
| Brass workers | 3 | 3 epidermoid |
| Metal milling machinists | 2 | 1 epidermoid<br>1 anaplastic |
| Metal buffer | 1 | Epidermoid |
| Metal chipper | 1 | ,, |
| Tool maker | 1 | ,, |
| Crane operator over molten metals | 1 | ,, |
| Iron fettler | 1 | Nil |
| Tin worker | 1 | ,, |

*Painters* (Table IV).—The table shows details of occupation and type of carcinoma. Lungs from one case were examined and histological sections from two others, all with carcinoma of main bronchus. Wynder and Graham (1951) suggest that there is some slight evidence for suspecting an increased incidence of lung cancer in this occupation.

TABLE IV.—*Painters* (3—*All with Carcinoma*)

| Occupation | Total | Carcinoma |
|---|---|---|
| House painters | 2 | 2 epidermoid |
| Railroad car painter | 1 | Squamous |

*Group IV : Industries with increased cancer incidence*

*Asbestos* (Table V).—Table V shows the mode of exposure and type of carcinoma present. Lungs from sixty unselected cases were examined, thirteen with associated carcinoma. Multiple sections were available in each case from areas of local and general fibrosis. There is general agreement in this country that asbestosis is associated with an increased cancer incidence. Gloyne (1951) found 14 per cent ; the *Annual Report of the Chief Inspector of Factories* (1955) gives it as 16 per cent ; and Doll (1955) as 14·2 per cent.

*Chromates.*—Lung sections from nine chromate workers were examined. Seven cases had developed carcinoma, six anaplastic and one epidermoid. These had all worked in an American plant extracting chromium compounds from iron chromite ore. Their length of exposure varied from four to twenty-five years. The incidence of respiratory carcinoma in this industry is twenty-nine times the

TABLE V.—*Asbestos* (60 ; 13 *with Tumours*)

| Occupation | Total | Carcinoma |
|---|---|---|
| Asbestos unspecified | 22 | 1 anaplastic<br>2 epidermoid |
| Weavers | 7 | 1 epidermoid |
| Miners | 6 | 1 squamous |
| Fabricating workers | 5 | 1 adenocarcinoma<br>1 endothelioma of pleura |
| Pipe makers | 4 | 1 epidermoid |
| Mill workers | 4 | 1 adenocarcinoma<br>1 alveolar cell tumour |
| Bagger | 3 | 1 anaplastic |
| Crushing room | 3 | 1 squamous<br>1 endothelioma of pleura |
| Carding room | 4 | — |
| Waste sorters | 2 | — |

crude mortality rate for all males in the United States (U.S. Public Health Service Publication, 1953). Machle and Gregorius (1948) state that 21 per cent of all deaths in the United States chromate-producing factories are due to carcinoma of the lung. There is doubt as to the occurrence of specific fibrosis in this occupation though bronchitis is common (US. Public Health Service Publication, 1953). With the exception of the two non-tumour cases material was limited to existing sections consisting mainly of tumour and not specifically areas of fibrosis.

*Nickel.*—Nickel workers are thought to show an increased incidence of lung cancer, the *Annual Report of the Chief Inspector of Factories* (1949) mentions eighty-two cases occurring between the years 1923 and 1948 inclusive. Lungs from three cases were available for study, one of which had developed a squamous carcinoma of a major bronchus. Multiple sections were examined from areas of fibrosis. The three men had worked in the same plant where nickel is extracted from the ore by the nickel carbonyl process. Cases 1 and 2 had no definite evidence of exposure to the gas nickel carbonyl ; Case 3, however, had one mild episode of nickel carbonyl poisoning one year before death. All workers had been exposed to mixed dust during refining of the ore.

### RESULTS

### *Age*

The overall inception rate of alveolar metaplasia typical or atypical, with or without carcinoma, may be seen in Fig. 2.

One case of berylliosis aged 7 is not included and cases in the eighth and ninth decade are grouped together because of their small number.

It is apparent that the incidence of alveolar metaplasia in all groups combined decreases with age.

### *Incidence*

The total population examined can be subdivided into those with and those without carcinoma, and according to occupation with and without carcinoma. The presence of alveolar metaplasia in each group was determined.

The crude rates of alveolar metaplasia in these sub-populations are not comparable owing to the differences in the age distributions of the various populations. When, by standardisation, we allow for such age variation, and so obtain

FIG. 2.—The overall inception rate of alveolar metaplasia.

the standardised inception rate, then a true comparison of the overall rates for each subgroup may be made (Table VI). Group III is the combination of all cases in each of its four subgroups. In Group IV the asbestos cases only are included as the ages of the chromate workers were not available and the number of nickel cases was very small (three).

TABLE VI.—*Standardised Inception Ratios (S.I.R.) for Alveolar Metaplasia*

(All subgroups combined = 100)

| | Without carcinoma | | | | | With carcinoma | | | | Rate for all groups per 100 |
|---|---|---|---|---|---|---|---|---|---|---|
| | Coal | Beryllium | Group III | Asbestos | Total | Coal | Group III | Asbestos | Total | |
| Number of persons | 100 | 45 | 22 | 38 | 205 | 30 | 10 | 7 | 47 | 41·4=100 |
| S.I.R = standardised rate expressed as % of rate for all groups | 38 | 112 | 200 | 150 | 90 | 91 | 188 | 167 | 136 | — |
| Standard error of above | 10·11 | 21·16 | 70·75 | 30·63 | 10·49 | 28·73 | 48·38 | 74·67 | 24·90 | — |

Table VI shows that the rate for all populations surveyed was 41·4 per 100 persons and for the total population without carcinoma it is 90 per cent of this as compared with 136 per cent for all persons with carcinoma. This difference, while not statistically significant, accounted for by the small number of cases with carcinoma, is somewhat suggestive of an increased incidence of alveolar metaplasia in cases with developed carcinoma.

The only group that shows a significant difference in the rate of alveolar metaplasia as compared to the rate for all cases in the survey is coal workers without carcinoma ; this rate is significantly low. That is, alveolar metaplasia in coal workers without carcinoma is less frequent than was found in all groups combined.

If one compares the rate of alveolar metaplasia in each group in the class without carcinoma with the rate for the whole class it is found that the difference in the rate for asbestosis approaches a significant level (difference $60 \pm 32$). The incidence of alveolar metaplasia in asbestosis without carcinoma tends to be greater than the incidence for the whole class without carcinoma. The difference in the other groups without carcinoma does not even approach a significant level.

Similarly, we can compare the rate for any one group in the class with carcinoma to the rate for the whole class, the difference is in no instance significant.

The rate of alveolar metaplasia in any one group without carcinoma compared to the rate for that group with carcinoma is in no instance significant. In coal workers the difference approaches significance. This suggests that the incidence of alveolar metaplasia in coal miners with carcinoma is higher than the incidence in coal miners without carcinoma.

Whilst determining the presence of alveolar metaplasia the extent of this change was also observed. It was found that irrespective of occupation or the presence of carcinoma it was most extensive in those cases with diffuse fibrosis. Two of the groups, berylliosis and asbestosis, show diffuse fibrosis as a constant feature and in these alveolar metaplasia when present was very extensive.

*Morphology*

In Group I (coal workers) the morphology of alveolar metaplasia was identical in the presence or absence of carcinoma. The metaplasia was not particularly related to areas of fibrosis, the involved air spaces being mainly perivascular, peribronchial, septal or subpleural. In no case did the metaplasia show evidence of malignancy, transition to malignancy or continuity with established tumour. The cells were mainly cuboidal, with minimal irregularity of size and shape, and a great dearth of mitosis. They were arranged in a single layer, with no papillary areas. The features were those constituting " typical " alveolar metaplasia (Fig. 3).

In Group II (berylliosis) the features were similar to Group I, typical alveolar metaplasia (Fig. 4).

Group III (oil, haematite, metal and paint workers) showed a similar appearance and distribution apparently unconnected and unrelated to carcinoma. One haematite miner with endothelioma of the pleura showed no typical alveolar metaplasia but many subpleural alveoli were lined by malignant cells in continuity with the tumour. This appearance (Fig. 5) closely resembles alveolar cell carcinoma but the tumour was thought to originate in the pleura.

In Group IV (the asbestos workers with alveolar metaplasia) with two exceptions, showed the typical variety. This was mainly at the margins of secondary lobules and unrelated to developed carcinoma. It is emphasised that atypical alveolar metaplasia was not present in any of the cases with developed carcinoma. The two exceptions which showed atypical metaplasia will be considered individually, as will the case of alveolar cell carcinoma.

*Case 27.*—Male, aged 53, asbestos miner 19 years. No carcinoma. Moderate degree of interstitial fibrosis with asbestos bodies. There was no evidence of infection or chronic venous congestion in the sections examined. The alveolar metaplasia was not extensive and was related to septa and areas of fibrosis. It was considered slightly atypical in that some areas showed more than one layer. The cells tended to be large with considerable hyperchromatism but no evidence of mitosis (Fig. 6). These spaces may be peripheral bronchioles and not alveoli but the epithelium is atypical for either.

*Case 32.*—Male, aged 62, asbestos pipe worker with mixed exposure to silica and asbestos. No carcinoma. Moderate degree of diffuse fibrosis with asbestos bodies, areas of caseous tuberculosis and classical whorled silicotic nodules. In one area, unrelated to tuberculosis or silicosis there was somewhat atypical alveolar metaplasia. Instead of the usual regular cuboidal epithelium, the cells varied in size, shape and staining and some can be seen desquamating into the alveolar spaces (Fig. 7). This slightly atypical appearance is probably associated with the oedema and represents formation of macrophages.

*Case 58.*—Female, age unknown, asbestos mill worker. Alveolar cell carcinoma. The lungs showed generalised fibrosis with scanty asbestos bodies. The alveolar spaces were lined by non-mucin-secreting columnar cells showing scanty mitosis (Fig. 8). In some areas these cells were multilayered. In the absence of an extrapulmonary primary neoplasm or carcinoma of main bronchus this condition is considered as alveolar cell carcinoma of the diffuse type, involving the whole lung. There was no metaplasia or evidence of a primary carcinoma in the major bronchi. The usual cuboidal alveolar metaplasia was absent.

Group IV, chromate workers, showed four instances of typical alveolar metaplasia, three in lungs with carcinoma of major bronchi and one without carcinoma. The epithelium in all cases was identical and consisted of a single layer of uniform cuboidal cells. There was no continuation or connection with the established bronchial tumours. Due to the paucity of material the relationship to fibrosis could not be evaluated.

Group IV, nickel workers, consisted of three cases which will be considered individually.

*Case 1.*—Male, aged 58, nickel worker 30 years. No exposure to nickel carbonyl. Chronic bronchitis 30 years. Epidermoid carcinoma. This man had an epidermoid carcinoma of the right upper lobe bronchus, a slight degree of interstitial fibrosis and " compensatory " emphysema. There was no evidence of alveolar (or bronchial) metaplasia.

*Case 2.*—Male, aged 34, nickel worker 4 years. No exposure to nickel carbonyl. No tumour. The lungs showed widespread fibrosis, bronchiectasis, bronchiolectasis and " cystic change ". The " cysts " were lined by epithelium designated as atypical alveolar metaplasia. This consisted of simple cuboidal cells and scattered bizarre giant-cells, some of which lay free in the air spaces (Fig. 9). The metaplasia was not considered malignant and was of the type sometimes associated with long-standing infection and fibrosis.

*Case 3.*—Male, aged 58, nickel worker 17 years. One mild attack nickel carbonyl poisoning one year before death. Alveolar cell carcinoma. The lungs showed widespread interstitial fibrosis, bronchiolectasis and " cystic " change. The " cysts " were lined by very atypical squamoid cells amounting to carcinoma (Fig. 10). As this epithelium was lining peripheral air passages in the absence of a primary tumour of main bronchus or other organ it was considered as an example of alveolar cell carcinoma. Typical alveolar cell metaplasia was to all intents absent and there was no transition from one to the other.

*Infection*

The relation of infection to the presence of alveolar metaplasia in the different groups (*a*) with carcinoma, (*b*) without carcinoma, may be seen in Table VII. Infection includes both pyogenic and tuberculous inflammation. The age incidence is not considered in this and the following Table VIII, so Group IV thus includes asbestos, chromates and nickel workers. The difference in the percentage of cases with and without infection was analysed. This reached a significant level in one instance, Group IV without carcinoma, i.e. those cases in Group IV with alveolar metaplasia, in the absence of carcinoma, show an increased incidence of infection. With this one exception infection does not apparently predispose to the development of alveolar metaplasia.

TABLE VII.—*The Relation of Infection to Presence of Alveolar Metaplasia*

| Occupational group | Alveolar + Metaplasia 0 | (a) With carcinoma: Number of cases | (a) With carcinoma: % infection | (b) Without carcinoma: Number of cases | (b) Without carcinoma: % infection |
|---|---|---|---|---|---|
| I | + | 10 | 90 | 13 | 61 |
| | 0 | 20 | 60 | 87 | 67 |
| II | + | — | — | 35 | 28 |
| | 0 | — | — | 17 | 47 |
| III | + | 16 | 81 | 8 | 100 |
| | 0 | 7 | 71 | 2 | 50 |
| IV | + | 10 | 50 | 34 | 82 |
| | 0 | 12 | 50 | 16 | 37 |

*Chronic venous congestion*

Table VIII shows the relationship of chronic venous congestion, including oedema, to the presence of alveolar metaplasia, (*a*) with carcinoma, (*b*) without carcinoma. The difference between the incidence of chronic venous congestion in those cases with and without alveolar metaplasia is in no instance significant. Chronic venous congestion does not therefore appear to predispose to alveolar metaplasia.

TABLE VIII.—*The Relation of Chronic Venous Congestion (Including Oedema) to Presence of Alveolar Metaplasia*

| Occupational group | Alveolar + Metaplasia 0 | (a) With carcinoma: Number of cases | (a) With carcinoma: % C.V.C. | (b) Without carcinoma: Number of cases | (b) Without carcinoma: % C.V.C. |
|---|---|---|---|---|---|
| I | + | 10 | 60 | 13 | 61 |
| | 0 | 20 | 30 | 87 | 48 |
| II | + | — | — | 35 | 40 |
| | 0 | — | — | 17 | 41 |
| III | + | 16 | 12 | 8 | 12 |
| | 0 | 7 | 0 | 2 | 50 |
| IV | + | 10 | 30 | 34 | 35 |
| | 0 | 12 | 33 | 16 | 18 |

DISCUSSION

The results show that the frequency of alveolar metaplasia decreases with age (Fig. 2). It thus does not parallel the increasing incidence of lung cancer with advancing age (Korteweg, 1951). This evidence is therefore against alveolar metaplasia being related to the development of lung cancer.

A comparison of the incidence of alveolar metaplasia in various occupations provides information as to whether the metaplasia is related to the development of carcinoma.

If alveolar metaplasia is premalignant then the frequency might be expected to be greater in the class " with carcinoma " than in the class " without carcinoma ". A statistical test shows that there is no significant difference in the standardised inception ratio of these two classes Similarly, if one considers a single occupational group the incidence might be expected to be greater in those cases with developed carcinoma than in those without carcinoma. The results show that the difference is not statistically significant. Thus, the association of fibrosis, alveolar metaplasia and carcinoma is no more frequent than the association of fibrosis and alveolar metaplasia. Another postulate was also tested : if alveolar metaplasia is premalignant then it might be expected to be commoner in occupations with a high incidence of cancer, e.g. asbestos workers, than in low cancer incident occupations, e.g. coal workers. The only results in favour of this postulate were that in the class " without carcinoma " the rate of alveolar metaplasia in asbestos workers tended to be greater than the rate for the whole class and that of coal workers was lower than the rate for all cases in the survey.

It is thought that the balance of evidence from incidence study does not favour a relationship between alveolar metaplasia and the development-of carcinoma.

The presence of alveolar metaplasia, with one exception, could not be correlated with infection or chronic venous congestion. The high incident tumour occupations, Group IV, without carcinoma showed a significantly high association of alveolar metaplasia with infection. It was important to assess these features as the incidence of alveolar metaplasia was expected to be increased in cases with carcinoma, and might have been accounted for by the frequent complication of infection. The evidence from this study suggest that with the one exception, infection and chronic venous congestion do not predispose to alveolar metaplasia. This is at variance with the work of Gazeyerli (1936) and Geever, Neuberger and Davies (1943).

If alveolar metaplasia is related to the development of carcinoma then one might expect to find them in histological continuity. In no section examined was there any evidence of continuity.

The morphology of alveolar metaplasia, with three exceptions was typical, with no suggestion of malignancy. Two of the atypical type occurred in asbestos workers and one in a nickel worker, but were not thought to be premalignant. One asbestos and one nickel worker showed alveolar cell carcinoma. In 278 cases examined there were thus only two instances of definite malignant disease arising in respiratory air passages. In no instance was there any evidence that the morphology of alveolar metaplasia was related to the presence of carcinoma of the bronchus, in fact atypical alveolar metaplasia did not occur in the presence of main bronchus carcinoma. There was also no transitional type of epithelium between alveolar metaplasia and bronchial or alveolar cell carcinoma. It is concluded that alveolar metaplasia on morphological grounds is not related to the development of carcinoma. This is in keeping with Gloyne's (1951) conclusion that he could not demonstrate any premalignant changes in asbestosis, and refutes Hutchinson (1952) who suggested that alveolar metaplasia is a precursor of alveolar cell carcinoma.

Barnard and Day (1937) thought that the finding of cuboidal metaplasia in alveoli adjacent to scars was accounted for by their relative fixity. This is partially supported by its frequent presence at the margins of secondary lobules, but the fact that alveolar metaplasia was seen adjacent to " scars " in only 14 per cent of coal workers strongly rebuts this theory. These scars presumably, are as fixed as any other scar. The evidence, thus tends to refute the " fixation " aetiology of alveolar metaplasia.

The hypothesis of Raeburn and Spencer (1953) that many hilar carcinomas may arise in the peripheral air passages appears questionable. In support of their theory they quote the findings of unsuspected microscopic tumours arising in peripheral scars. The results of this investigation do not support their theory. It may be stressed that no localised tumour was found in peripheral scars in any of the industrial lungs examined. It is indeed surprising that inhaled carcinogens do not seem to stimulate the 100 square metres of peripheral epithelium but rather choose the few square centimetres of major bronchi. The hypothesis of Macklin (1956) of carcinogens concentrating in the major bronchi by the action of a " mucus raft " is most attractive. Neuberger and Geever (1942) in an extensive review of the literature up to 1940 state that the majority of authors do not incriminate diffuse fibrosis as a cause of alveolar cell neoplasia. The present findings show that diffuse fibrosis favours the production of benign cuboidal alveolar metaplasia but not alveolar cell neoplasia. This does not imply that diffuse fibrosis is not a " co-carcinogenic " factor in the development of lung cancer. It may be the factor explaining the greater cancer incidence in asbestosis as compared to coal workers.

### SUMMARY

The epithelium lining respiratory portions of the lung adjacent to areas of fibrosis was examined in 278 cases, and its relationship to the development of carcinoma ascertained.

Cases were selected from occupations with different incidence of lung cancer varying from low to high and included coal, beryllium, oil, tar, haematite, metal, paint, asbestos, chromate and nickel workers. In each occupation, when available, cases with and without carcinoma were examined.

Alveolar metaplasia was the term applied to cuboidal epithelium lining respiratory air spaces, and was subdivided into typical and atypical.

The overall inception rate of alveolar metaplasia decreased with age.

Alveolar metaplasia in the lungs from occupations with a high incidence of cancer did not show any conclusive difference in frequency or morphology from that seen in occupations with a low incidence of cancer.

The presence of an established carcinoma was not related to the incidence or morphology of alveolar metaplasia.

Atypical alveolar metaplasia occurred in only three cases, two asbestos workers and one nickel worker. The three cases, though atypical, were not malignant or thought to be premalignant. In the 278 cases examined there were only two carcinomas, alveolar cell, thought to arise in the respiratory air passages, one in an asbestos worker and one in a nickel worker.

Alveolar metaplasia was not particularly related to isolated scars. Irrespective of occupation diffuse fibrosis of the lung predisposed to alveolar metaplasia. " Fixity " of lung scars was not thought to predispose to alveolar metaplasia. No localised tumour was found in any of the hundreds of peripheral scars examined.

The frequency of alveolar metaplasia is not related to the presence of infection or chronic venous congestion.

### CONCLUSION

Cuboidal epithelium, alveolar metaplasia, as seen in alveoli and peripheral respiratory air passages, is thought to be unrelated to the development of pulmonary carcinoma.

I am indebted to the British Empire Cancer Campaign for an American-British Exchange Fellowship, 1954–55.

I wish to express my warm thanks to many American pathologists and clinicians for their kind interest and for access to their material and records, in particular Drs. A. A. Liebow, B. Castleman, G. W. H. Schepers, A. J. Vorwald, H. L. Hardy and G. Lindskog.

My thanks are due to Professor J. Gough for his valuable advice and criticism. Also to Dr. Lewis Fanning for the statistical analyses and to Dr. W. R. L. James for access to his material.

### REFERENCES

'Ann. Rep. Chief Insp. Factories for 1948'.—(1949) p. 95. London (H.M. Stationery Office).
'Ann. Rep. Chief Insp. Factories for 1954'.—(1955) p. 190. London (H.M. Stationery Office).
BARNARD, W. G. AND DAY, T. D.—(1937) *J. Path. Bact.*, **45**, 62.
BONSER, G. M., FAULDS, J. S. AND STEWART, H. J.—(1955) *Amer. J. clin. Path.*, **25**, 126.
DOLL, R.—(1955) *Brit. J. industr. Med.*, **12**, 81.
DUTRA, F. R.—(1948) *Amer. J. Path.*, **24**, 1137.
*Idem*, LARGENT, E. J. AND ROTH, J. L.—(1951) *Arch. Path.*, **51**, 473.
GAZAYERLI, M.—(1936) *J. Path. Bact.*, **43**, 357.
GEEVER, E. F., NEUBERGER, K. T. AND DAVIES, C. L.—(1943) *Amer. J. Path.*, **25**, 913.
GLOYNE, S. R.—(1952) *Lancet*, i, 810.
GOUGH, J.—(1947) *Occup. Med.*, **4**, 86.
HARDY, H. L. AND TABERSHAW, I. R.—(1946) *J. industr. Hyg.*, **28**, 197.
HEPPLESTON, A. G.—(1951) *Arch. industr. Hyg.*, **4**, 270.
HUEPER, W. C.—(1942) 'Occupational Tumors and Allied Diseases', p. 420. Springfield, Illinois (Thomas). (1956) *Dis. Chest*, **30**, 141.
HUTCHINSON, H. E.—(1952) *Cancer*, **5**, 884.
JAMES, W. R. L.—(1955) *Brit. J. industr. Med.*, **12**, 87.
KENNAWAY, N. M. AND KENNAWAY, E. L.—(1936) *J. Hyg.*, **36**, 236.
KING, L. S.—(1954) *Arch. Path.*, **58**, 59.
KORTEWEG, R.—(1951) *Brit. J. Cancer*, **5**, 21.
MACHLE, W. AND GREGORIUS, F.—(1948) *Publ. Hlth. Rep., Wash.*, **63**, 1114.
MACKLIN, C. C.—(1956) *J. thorac. Surg.*, **31**, 238.
McLAUGHLIN, A. I. G. *et al.*—(1950) 'Industrial Lung Diseases of Iron and Steel Foundry Workers'. London (H.M. Stationery Office).

NEUBERGER, K. T. AND GEEVER, E. F.—(1942) *Arch. Path.*, **83**, 551.
PRIOR, J. T.—(1953) *Amer. J. Path.*, **29**, 703.
RAEBURN, C. AND SPENCER, H.—(1953) *Thorax*, **8**, 1.
'Saranac Symposium on Pneumoconiosis, 6th Symposium, 1947'.—(1950) edited by A. J. Vorwald, Paul B. Hoeber, inc.
SCHEPERS, G. W. H., DURKAN, T. M., DELAHANY, A. B. and CREADON, F. T. (1957) *A. M. A. Arch. industr. Hlth.*, **15**, 32.
SPENCER, H. AND RAEBURN, C.—(1954) *J. Path. Bact.*, **67**, 187.—(1956) *Ibid.*, **71**, 145.
STEWART, M. J. AND FAULDS, J. S.—(1934) *Ibid.*, **39**, 233.
'U.S. Public Health Service Publication No. 192'.—(1953).
VORWALD, A. J.—(1950) 'Saranac Symposium on Pneumoconiosis, 6th Symposium, 1947'.
WOMACK, N. A. AND GRAHAM, E. A.—(1941) *Amer. J. Path.*, **17**, 645.
WYNDER, E. L. AND GRAHAM, E. A.—(1951) *Arch. industr. Hyg.*, **4**, 221.